# Comparison of the Effects of Articaine and Lidocaine Anesthetics on Blood Pressure after Maxillary Infiltration Technique: A Triple-Blind Randomized Clinical Trial

**DOI:** 10.1155/2021/8894160

**Published:** 2021-08-26

**Authors:** Amirhossein Moaddabi, Parisa Soltani, Maryam Zamanzadeh, Kamran Nosrati, Mojtaba Mollamirzaei, Mariangela Cernera, Gianrico Spagnuolo

**Affiliations:** ^1^Department of Oral and Maxillofacial Surgery, Dental Research Center, Mazandaran University of Medical Sciences, Sari, Iran; ^2^Faculty of Dentistry, Mazandaran University of Medical Sciences, Sari, Iran; ^3^Department of Oral and Maxillofacial Radiology, Dental Implants Research Center, Dental Research Institute, School of Dentistry, Isfahan University of Medical Sciences, Isfahan, Iran; ^4^Department of Oral and Maxillofacial Pathology, School of Dentistry, Mazandaran University of Medical Sciences, Sari, Iran; ^5^Department of Oral and Maxillofacial Surgery, School of Dentistry, Babol University of Medical Sciences, Babol, Iran; ^6^Dental Students' Research Committee, School of Dentistry, Mazandaran University of Medical Sciences, Sari, Iran; ^7^Department of Neurosciences, Reproductive and Odontostomatological Sciences, University of Naples “Federico II”, Naples, Italy; ^8^Institute of Dentistry, I. M. Sechenov First Moscow State Medical University, Moscow 119435, Russia

## Abstract

**Background:**

Many dental procedures begin with local anesthesia. Subsequent increase in blood pressure in healthy individuals commonly occurs and may be affected by several factors such as mental and physical stress, painful stimuli, and action of catecholamines present in local anesthetic solutions. The aim of the present study is to compare the effects of 4% articaine with 1 : 100000 epinephrine and 2% lidocaine with 1 : 80000 epinephrine on blood pressure after maxillary infiltration technique.

**Materials and Methods:**

In this randomized clinical trial, 102 patients were randomly assigned into two groups. One group received 4% articaine with 1 : 100000 epinephrine and the other group received 2% lidocaine with 1 : 80000 epinephrine for local maxillary infiltration. Systolic and diastolic blood pressure of both groups was determined twice: once before anesthetic injection and once 10 minutes after injection. The data were statistically analyzed using descriptive statistics, Shapiro–Wilks test, Levene test, chi-square test, independent *t*-test, and paired *t*-test.

**Results:**

The mean systolic blood pressure after anesthetic injection in the articaine and lidocaine groups was 125.00 ± 5.67 and 123.16 ± 6.417 mmHg, respectively, showing no statistically significant difference (*p*=0.127). The mean diastolic blood pressure after injection was 85.02 ± 7.331 in the articaine group and 81.35 ± 12.815 mmHg in the lidocaine group. These values show no statistically significant difference (*p*=0.080). In both groups, the mean systolic and diastolic blood pressures have increased significantly (*p* < 0.001).

**Conclusion:**

Articaine can be regarded as a suitable alternative for lidocaine for maxillary local infiltration, as no significant difference was observed between the effects of the two anesthetic solutions on blood pressure.

## 1. Introduction

Many dental procedures are preceded with local anesthesia. Anesthetic drugs are among the most common drugs used in dentistry. It has been estimated that annually more than 300 million cartridges of local anesthetics are used by dentists in the United States [1].

Increased blood pressure is common after injection of local anesthetic drugs [2]. The amount of this increase is modified by several factors such as psychological and physical stress, painful stimuli, and catecholamines present in dental anesthetic solutions [3, 4]. Identification of increased blood pressure is important as fatal subarachnoid hemorrhage and excessive bleeding from dental surgery has been reported [5, 6]. Studies have shown that the rise in blood pressure during dental surgery results primarily from activation of the sympathetic nervous system [7, 8].

A variety of dental anesthetic drugs are available for dental purposes [9]. Lidocaine and articaine are among these agents [10]. Lidocaine is an amide anesthetic drug routinely used in dentistry [11]. This agent is metabolized in the liver by multipurpose microsomal oxidase enzymes to monoethyl glycine and its derivatives. Its excretion from the body is via the kidneys. Less than 10% of the drug is excreted unchanged and more than 80% is excreted as different metabolites [12]. Articaine is one of the amide anesthetic agents and its pharmacological characteristics result in several advantages [13, 14]. In addition to the characteristics of most amide anesthetics, articaine has an aromatic ring which enhances its protein bindings and enables higher penetration and diffusion in the tissues [12].

Several studies have attempted to compare the hemodynamic effects of different anesthetic solutions. Abu-Mostafa et al. evaluated the hemodynamic effects of articaine and lidocaine solutions with different epinephrine concentrations and concluded that diastolic blood pressure, heart rate, and O_2_ saturation after anesthesia and exodontia showed no significant difference among the groups. However, since the articaine solution with the least concentration of vasoconstrictor had the smallest effect on systolic blood pressure, it was considered safer for anesthesia before dental extraction [15]. In another study, Stella et al. evaluated the hemodynamic variations of lidocaine and articaine solutions in impacted third molar surgery and concluded that these two anesthetics lead to similar hemodynamic effects [16].

Aside from type of anesthetic drugs, the technique of the injection is important for appropriate anesthesia [17, 18]. The routine injection technique for anesthesia of maxillary teeth is infiltration technique. The literature regarding comparison of maxillary infiltration of lidocaine and articaine anesthetic solution is scarce. Therefore, the aim of the present study was to evaluate the effects of 4% articaine with 1 : 100000 epinephrine on blood pressure after maxillary infiltration technique.

## 2. Materials and Methods

This triple-blind randomized clinical trial was approved by the Ethical Committee of Mazandaran University of Medical Sciences (code IR.MAZUMS.REC.1398.544). The protocol for this trial was registered in the Iranian Registry of Clinical Trials (#IRCT20200724048188N1). The trial was performed on candidates for nonsurgical extraction of posterior maxillary teeth attending Department of Oral and Maxillofacial Surgery of Sari Dentistry School, Iran, from October 2018 to July 2019. All patients signed the informed consent form designed in accordance with the principles of the Helsinki Declaration prior to enrollment and were free to withdraw from the study at any stage.

Sample size was calculated based on 95% confidence interval, 90% power as 88 (44 in each experimental group). Considering 15% sample loss, the sample size was increased to 102 (51 in each experimental group).

Inclusion criteria were class 1 American Society of Anesthesiologists patients aged 18 to 55 years, not taking any medication, no contraindication for administration of epinephrine, containing anesthetics, nonsmoker patients, without dental phobia, without excessive pain or swelling on admission, and not being pregnant. Patients who experienced dental emergencies after injection were excluded from the study.

**Figure 1:**
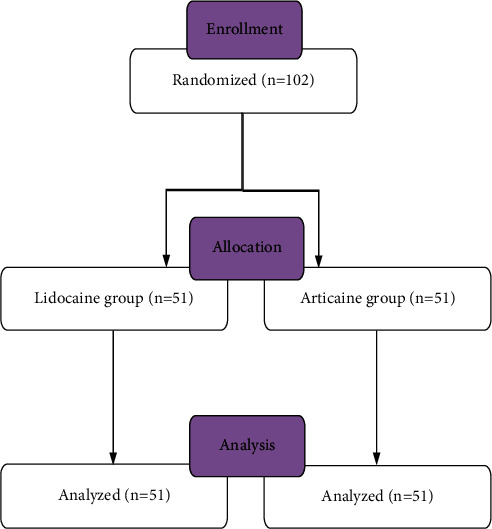
Flow diagram of the study participants.

The patients were randomly assigned using computer-generated random numbers into two groups: lidocaine group and articaine group (Figure 1). The patients and operators were blind to the experimental groups in all stages of the research study. Only one researcher, who was present in the surgical room but was unrelated to the experiments and measurements, was aware of the experimental group of each patient.

Demographic characteristics of the patients were recorded. All patients were positioned semisupine in the dental chair. Systolic and diastolic blood pressures were measured after a 15-minute rest, while the patient's hand was straight at the heart level using a manual sphygmomanometer (Pooyateb, Tehran, Iran). Prior to the injection, the depth of the vestibule at the region was dried with gauze. Then, benzocaine anesthetic gel was applied to the area of injection for 3 minutes. Cartridges of 4% articaine with 1 : 100000 epinephrine and 2% lidocaine with 1 : 80000 epinephrine were used. The anesthetic cartridges were covered with a black tape by a researcher so that the operator does not know the type of the anesthetic drug. The cartridges were placed in an aspirating syringe with a 27-gauge short needle. Two-thirds of the anesthetic solution was infiltrated in the depth of the buccal vestibule of the tooth that was planned for extraction in 40 seconds. An additional 0.3 ml of the anesthetic agent was also injected in the palatal mucosa 1-2 mm below the free gingiva. All the injections were performed by an adequately trained dental student under supervision of an oral and maxillofacial surgeon. 10 minutes after injection, systolic and diastolic blood pressures were measured once again with the same method. The operators tried to control their verbal communication and overall environment of the room for all patients. Following the injection procedure, nonsurgical extraction was performed according to the preexisting treatment plan.

The statistician was blind to the experimental groups. Demographic characteristics of the patients were evaluated using descriptive statistics and were represented as means, standard deviations, medians, and frequency percentages. Prior to statistical analysis, normal distribution of the data and equality of variances were assessed using Shapiro–Wilk test and Levene test, respectively. To compare the sex distribution of the two groups, the chi-square test was used. To compare the mean systolic and diastolic blood pressures before and after anesthetic injection between the two groups, the independent *t*-test was used. To compare the mean systolic and diastolic blood pressures before and after injection in each group, the paired *t*-test was used. Statistical analysis was performed using Statistical Package for the Social Sciences (SPSS, v 16, IBM, IL, USA).

## 3. Results

102 consecutive patients enrolled in the study; 51 in each experimental group. The lidocaine and articaine groups were consisted of 26 males and 25 females and 27 males and 24 females, respectively. The sex distribution in the two groups was not significantly different (*p*=0.843). Mean age of the study participants in the lidocaine and articaine groups was 38.24 ± 2.080 and 39.63 ± 1.969, respectively. Mean age of the participants was not statistically different between the two experimental groups (*p*=0.602).

Mean systolic and diastolic blood pressures before anesthetic injection were not significantly different between lidocaine and articaine groups (*p*=0.540 and *p*=0.471, respectively). Moreover, mean systolic and diastolic blood pressures after anesthetic injection did not have a statistical significant difference between lidocaine and articaine groups (*p*=0.127 and *p*=0.080, respectively). However, the increase in systolic and diastolic blood pressures of the patients in each experimental group was statistically different (*p* < 0.001) (Table 1).

## 4. Discussion

In the present study, lidocaine and articaine both increased systolic and diastolic blood pressure after maxillary infiltration. However, these drugs were not significantly different in increasing the blood pressure. Similarly, Abu-Mostafa et al. found the same trend of significant increase in their study [15]. This increase can be a result of presence of vasoconstrictor agents in the anesthetic solution, as well as painful stimulus of needle injection and tissue manipulation. In a study performed by Rathi et al., mean systolic and diastolic blood pressures were not significantly different after lidocaine or articaine infiltration in children [19]. The difference between their findings and the findings of the current study can be attributed to differences in physiological features between children and adults and differences in the response and sensitivity of their cardiovascular system. Shah et al., in their study in children, found that changes in systolic and diastolic blood pressure is not significantly different between lidocaine and articaine groups. However, because the pain scale was higher in the lidocaine group, articaine can be a good alternative for lidocaine for dental anesthesia [20].

Epinephrine is used in dental anesthetic solutions as a vasoconstrictor agent for increasing depth and duration of the induced local anesthesia. This agent has various hemodynamic effects. Its alpha-adrenergic effects lead to peripheral vasoconstriction, and its beta-constriction increases heart constrictions and vasodilation is muscles. Therefore, epinephrine increases blood pressure and pulse rate and decreases blood pressure [21, 22]. Akinmoladun et al. compared the adverse effects of epinephrine-containing anesthetics and anesthetic agents without vasoconstrictor and found that no additional effect is caused in the group receiving epinephrine. They concluded that epinephrine-containing anesthetic solutions are preferred for surgical procedures [23]. In the present study, the epinephrine contents of lidocaine and articaine solutions were different: 1 : 100000 in articaine solution and 1 : 80000 in lidocaine solutions. Therefore, patients in the lidocaine group received higher doses of epinephrine. Although due to the small volume of the anesthetic drug in dental cartridges, the difference between epinephrine levels of the two anesthetic solutions is insignificant, and it is generally preferable to use epinephrine as low as possible.

No complication related to anesthetic injection, including facial edema, infection, or paresthesia, was observed in the present study. Malamed et al. reported that the most frequent adverse events following the use of articaine were headache, facial edema, infection, gingivitis, and paresthesia. However, due to the low frequency of such events, they concluded that articaine is a safe drug for clinical application [24].

This study had some limitations. Some of the factors affecting the patients' blood pressure, such as emotional status or time of the day, were not controlled. Future studies are needed to clarify the effects of various contributing factors in hemodynamic changes after injection of different anesthetic agents.

## 5. Conclusion

Articaine can be regarded as a suitable alternative for lidocaine for maxillary local infiltration, as no significant difference was observed between the effects of the two anesthetic solutions on blood pressure.

## Figures and Tables

**Table 1:** Mean values of systolic and diastolic blood pressures before and after lidocaine and articaine injection.

	Experimental group				*p*-value
	Lidocaine		Articaine		
	Mean (mmHg)	SD	Mean (mmHg)	SD	
SBP1	111.10	12.135	112.29	6.754	0.540
DBP1	77.71	12.082	77.71	6.607	0.471
SBP2	123.16	6.417	125.00	5.657	0.127
DBP2	81.35	12.815	85.02	7.331	0.080

SBP1: systolic blood pressure before anesthetic injection; SBP2: systolic blood pressure after anesthetic injection; DBP1: diastolic blood pressure before anesthetic injection; DBP2: diastolic blood pressure after anesthetic injection.

## Data Availability

The data used to support the findings of this study have been deposited in the Mendeley Data repository (10.17632/vg9sgd3nvc.1).

## References

[1] Malamed S. F. (2019). *Handbook of Local Anesthesia*.

[2] Matsumura K., Miura K., Takata Y. (1998). Changes in blood pressure and heart rate variability during dental surgery. *American Journal of Hypertension*.

[3] Tsuchihashi T., Takata Y., Kurokawa H. (1996). Blood pressure response during dental surgery. *Hypertension Research*.

[4] Abraham-Inpijn L., Borgmeijer-Hoelen A., Gortzak R. A. (1988). Changes in blood pressure, heart rate, and electrocardiogram during dental treatment with use of local anesthesia. *The Journal of the American Dental Association*.

[5] Okada Y., Suzuki H., Ishiyama I. (1989). Fatal subarachnoid haemorrhage associated with dental local anaesthesia. *Australian Dental Journal*.

[6] Niamtu J. (2001). Near-fatal airway obstruction after routine implant placement. *Oral Surgery, Oral Medicine, Oral Pathology, Oral Radiology & Endodontics*.

[7] Nakamura Y., Matsumura K., Miura K., Kurokawa H., Abe I., Takata Y. (2001). Cardiovascular and sympathetic responses to dental surgery with local anesthesia. *Hypertension Research*.

[8] Jeon Y., Shim J., Kim H. (2020). Junctional rhythm with severe hypotension following infiltration of lidocaine containing epinephrine during dental surgery. *Journal of Dental Anesthesia and Pain Medicine*.

[9] Mathur V. P., Kalra G. (2020). Insight to newer agents and methods for local anesthesia in pediatric dentistry. *The Indian Journal of Pediatrics*.

[10] Brandt R. G., Anderson P. F., McDonald N. J., Sohn W., Peters M. C. (2011). The pulpal anesthetic efficacy of articaine versus lidocaine in dentistry. *The Journal of the American Dental Association*.

[11] Bahar E., Yoon H. (2021). Lidocaine: a local anesthetic, its adverse effects and management. *Medicina*.

[12] Malamed S. F. (2019). *Handbook of Local Anesthesia-E-Book*.

[13] Colombini B. L., Modena K. C. S., Calvo A. M. (2006). Articaine and mepivacaine efficacy in postoperative analgesia for lower third molar removal: a double-blind, randomized, crossover study. *Oral Surgery, Oral Medicine, Oral Pathology, Oral Radiology & Endodontics*.

[14] Martin E., Nimmo A., Lee A., Jennings E. (2021). Articaine in dentistry: an overview of the evidence and meta-analysis of the latest randomised controlled trials on articaine safety and efficacy compared to lidocaine for routine dental treatment. *BDJ Open*.

[15] Abu-Mostafa N., Al-Showaikhat F., Al-Shubbar F., Al-Zawad K., Al-Zawad F. (2015). Hemodynamic changes following injection of local anesthetics with different concentrations of epinephrine during simple tooth extraction: a prospective randomized clinical trial. *Journal of Clinical and Experimental Dentistry*.

[16] Stella P. E. M., Falci S. G. M., Coelho V. S., dos-Santos C. R. R. (2018). Hemodynamic behavior in third molar surgeries using lidocaine or articaine. *International journal of odontostomatology*.

[17] Evans G., Nusstein J., Drum M., Reader A., Beck M. (2008). A prospective, randomized, double-blind comparison of articaine and lidocaine for maxillary infiltrations. *Journal of Endodontics*.

[18] Pozos-Guillén A., Loredo-Cruz E., Esparza-Villalpando V., Martínez-Rider R., Noyola-Frías M., Garrocho-Rangel A. (2020). Pain and anxiety levels using conventional versus computer-controlled local anesthetic systems in pediatric patients: a meta-analysis. *Journal of Clinical Pediatric Dentistry*.

[19] Rathi N. V., Khatri A. A., Agrawal A. G., M S. B., Thosar N. R., Deolia S. G. (2019). Anesthetic efficacy of buccal infiltration articaine versus lidocaine for extraction of primary molar teeth. *Anesthesia Progress*.

[20] Shah A. (2018). *Comparing the Effectiveness of Articaine Mandibular Infiltration vs. Lidocaine IANB in Pediatric Patients*.

[21] Brown R. S., Rhodus N. L. (2005). Epinephrine and local anesthesia revisited. *Oral Surgery, Oral Medicine, Oral Pathology, Oral Radiology & Endodontics*.

[22] Pallasch T. J. (1998). Vasoconstrictors and the heart. *Journal of the California Dental Association*.

[23] Akinmoladun V. I., Okoje V. N., Akinosun O. M., Adisa A. O., Uchendu O. C. (2013). Evaluation of the haemodynamic and metabolic effects of local anaesthetic agent in routine dental extractions. *Journal of Maxillofacial and Oral Surgery*.

[24] Malamed S. F., Gagnon S., Leblanc D. (2001). Articaine hydrochloride: a study of the safety of a new amide local anesthetic. *The Journal of the American Dental Association*.

